# Twin testosterone transfer hypothesis and the second‐to‐fourth digit ratio in females of same‐sex and opposite‐sex twin pairs: An exploratory study

**DOI:** 10.14814/phy2.70207

**Published:** 2025-01-22

**Authors:** Moses Banyeh, Martha Nyewie, Abdul‐Rafik Abdulai, Charles Nkansah, Gabriel Abbam, Thea Kangkpi, Kervin Edinam Zogli, Shafiat Omotoyosi Shittu, David Bure, Romarick Kofi Wemegah, Mikail Ihsan Azindow, Suleman Yakubu, Musah Seidu, Mohammed Madde Baba, Elisha Essoun, Nancy Owireduwaa, Augusta S. Kolekang

**Affiliations:** ^1^ Department of Biomedical Laboratory Science University for Development Studies Tamale Ghana; ^2^ Department of Public Health, Community Nurses Training School Ministry of Health Tamale Ghana; ^3^ Department of Histology and Histotechnology University for Development Studies Tamale Ghana; ^4^ Department of Haematology University for Development Studies Tamale Ghana; ^5^ Department of Infectious Diseases University for Development Studies Tamale Ghana; ^6^ Department of Immunology and Immunodiagnostics University for Development Studies Tamale Ghana; ^7^ Department of Population and Reproductive Health University for Development Studies Tamale Ghana; ^8^ Department of Epidemiology, Biostatistics and Disease Control University for Development Studies Tamale Ghana

**Keywords:** digit ratios, female, Ghana, humans, testosterone, twins

## Abstract

The twin testosterone transfer (TTT) hypothesis posits that females with male co‐twins (opposite‐sex, OS) might develop male‐typical traits due to higher prenatal testosterone exposure. This study explored whether females of OS have lower 2D:4D digit ratios and higher testosterone levels compared to females of same‐sex (SS) twin pairs. Conducted in Tamale from January to December 2022, the study included 40 participants aged 18–27 years: 10 males of OS, 10 females of OS, and 20 females of SS twin pairs. Digit ratios (2D:4D) and serum testosterone levels were measured using computer‐assisted analysis and ELISA, respectively. Results showed no significant differences in 2D:4D ratios between females of OS and SS twin pairs for either the right hand (0.960 ± 0.049 vs. 0.955 ± 0.042; *p* = 0.766) or the left hand (0.966 ± 0.048 vs. 0.968 ± 0.047; *p* = 0.908). Serum testosterone levels were lower in females of OS than females of SS twin pairs (0.4 ± 0.1 vs. 0.67 ± 0.34 nmol/L; *p* = 0.013), but this result was not significant after multiple testing corrections (*p* > 0.050). The findings indicate that the TTT hypothesis may not apply, or its effects on digit ratios and testosterone levels in females of OS twin pairs are weak and not statistically significant. Further studies involving larger samples are however, recommended.

## 1 INTRODUCTION

The twin testosterone transfer (TTT) hypothesis suggests that females with male co‐twins may exhibit traits that are more typically male, on average, than females with female co‐twins including cognitive, motor, behavioural, and physical characteristics (Tapp et al., 2011). This phenomenon is attributed to their exposure to relatively higher prenatal androgen from their male co‐twins (Ahrenfeldt et al., 2020). In the context of humans, there are two conceivable mechanisms or pathways for prenatal testosterone transfer between foetuses: the maternal–foetal and the foetal–foetal routes. Support for both mechanisms has been derived from animal studies (Ryan & Vandenbergh, 2002). Pregnant animals receiving exogenous testosterone injections during pregnancy showed increased circulating testosterone and masculinized behavioural traits in their offspring. Additionally, female foetuses situated between two male foetuses tend to exhibit masculinization (Ahrenfeldt et al., 2020; Ryan & Vandenbergh, 2002; Tapp et al., 2011).

Amniotic fluid can permeate the foetal skin and the placenta until the 18th week of gestation, a period coinciding with the potential peak of testosterone production in males (Ahrenfeldt et al., 2015). Nevertheless, human studies provide stronger support for the foetal–foetal route of prenatal testosterone transfer, as there is no observed correlation between maternal and amniotic fluid testosterone (Ahrenfeldt et al., 2020; Rodeck et al., 1985; Tapp et al., 2011). Testosterone, the most biologically potent androgenic hormone, is produced by Leydig cells in the developing testes of males approximately 6 weeks after conception, preceding ovarian testosterone production in developing females (Ahrenfeldt et al., 2020; Bütikofer et al., 2019; Mitsui et al., 2015). Peak prenatal testosterone (PT) production in male foetuses occurs between weeks 8 and 24 of gestation, with a specific peak between weeks 8 and 18. This period aligns with the critical phase of foetal brain development in humans (Ahrenfeldt et al., 2020). According to the organizational hypothesis, prenatal androgen exposure has a lasting impact on brain lateralization, leading to the development of male‐ or female‐typical phenotypes in cognition, spatial ability, handedness, sporting ability, and behavioural traits, albeit this may be weak (Breedlove, 2010; Crewther et al., 2022). Additionally, prenatal hormone exposure may exert a defeminizing or demasculinizing effect by suppressing female‐ or male‐typical traits, respectively (Tapp et al., 2011).

A commonly observed trait in humans that is linked to prenatal testosterone exposure is the second‐to‐fourth digit (2D:4D) ratio. This ratio is considered a potential indicator of prenatal testosterone (PT) exposure, with males generally exhibiting a lower ratio than females in many populations (Manning et al., 2022). The right hand tends to be more sensitive to the effects of prenatal testosterone than the left hand, and a negative right–left hand difference between the 2D:4D may also suggest prenatal testosterone exposure although this may not be universally accepted. The masculinizing impact of prenatal testosterone on the 2D:4D ratio is believed to occur within a specific timeframe during foetal development, likely before the 14th week of gestation (Manning & Fink, 2018; Zheng & Cohn, 2011).

Earlier studies have suggested a synergistic interaction between prenatal testosterone (PT) and prenatal oestrogen (PE) in shaping the 2D:4D ratio. Evidence for the masculinizing or feminizing effects of prenatal hormone exposure is particularly apparent in conditions involving abnormal steroidogenesis. For example, congenital adrenal hyperplasia (CAH) is associated with androgen excess, while Klinefelter's syndrome or complete androgen insensitivity syndrome involves androgen deficiency or insensitivity (Chang et al., 2015; Manning et al., 2013; Ökten et al., 2002).

Previous studies that have examined the twin testosterone transfer hypothesis about the 2D:4D ratio have been inconclusive (Cohen‐Bendahan et al., 2004; Medland, Loehlin, & Martin, 2008; Voracek & Dressler, 2007). The twin testosterone transfer hypothesis carries implications for the health of female twins. Testosterone, a crucial factor in hematopoiesis and biochemical processes, contributes to sex‐dependent variabilities in reference ranges (Bachman et al., 2014). Conditions such as cancer, infertility, and endometriosis have been linked to prenatal hormone exposure (Bunevicius, 2018). If the TTT holds, females of opposite‐sex twin pairs are anticipated to exhibit male‐typical testosterone levels and a second‐to‐fourth digit ratio (2D:4D) differing from females of same‐sex females. However, this hypothesis remains untested in the Ghanaian population.

Ethnic or ancestral diversity in research is vital for the verifiability or generalizability of research outcomes due to variabilities in genetic and environmental factors. A review of available literature, evaluating the TTT in females of same‐ and opposite‐sex twin pairs by Tapp et al. (2011), included 40 studies comprising behavioural differences (12), perceptual and cognitive differences (7) and physiological and morphological differences (21). However, none of these 40 primary studies was conducted in an endogenous Black‐African population. Moreover, only three studies were focused on the 2D:4D ratio and TTT at the time of the review (Medland, Loehlin, Willemsen, et al., 2008; van Anders et al., 2006; Voracek & Dressler, 2007). Even though this study was performed on a small sample size, it adds valuable information since there is, a lack of existing research in non‐European countries, particularly indigenous Black‐African populations. There is an apparent lack of ancestral diversity regarding the TTT and the 2D:4D ratio which warrant more research, particularly in non‐European populations.

## 2 MATERIALS AND METHODS

### 2.1 Research design and participants' demographics

Conducted as a cross‐sectional study spanning from January to September 2022, this investigation took place in Tamale, the largest metropolitan city in the northern region of Ghana. Tamale is characterized by its diverse population, with the major cultural group being the Dagomba, which is part of the broader linguistic group in northern Ghana known as the Mole‐Dagomba. The designation of the participants into cultural groups is just self‐reported affiliations and not an indication of a distinct ethnicity or special characteristics that may pose a potential bias or confounding in the results. Since twin populations are usually smaller in the general population, it was a challenge finding participants, so a convenient sampling technique was adopted. The study included twin pairs encompassing 10 (25.0%) males of opposite‐sex (OS) twin pairs, 10 (25.0%) females of OS twin pairs, and 20 (50.0%) females of same‐sex (SS) twin pairs. The zygosity of the same‐sex twins was not established as the study focused on only the sex of the twin pair. Participants were selected without a documented history of finger fractures, hormonal abnormalities, or chronic diseases that could significantly impact the study outcomes.

### 2.2 Data collection and measurements

**FIGURE 1:**
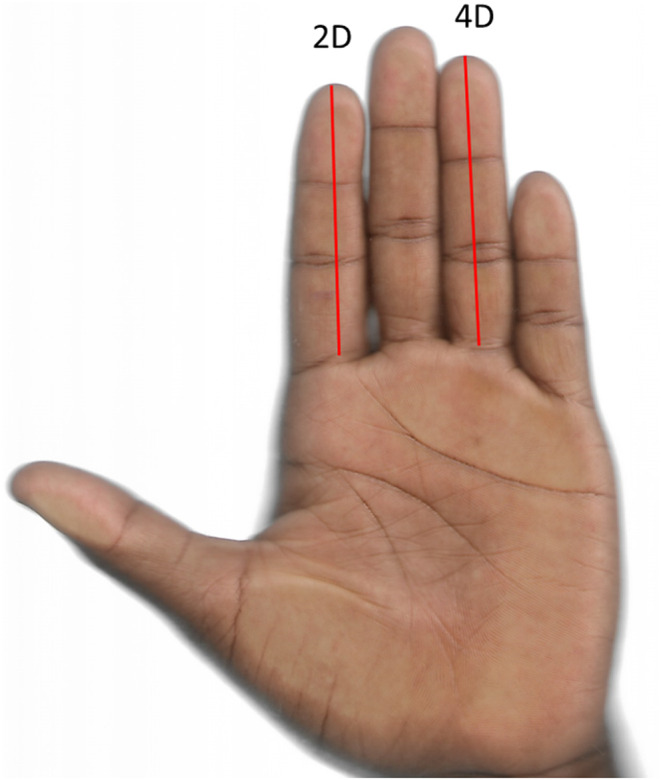
The palmar surface of the hand shows the length of the second digit (2D) and fourth digit (4D). Each digit was measured from the most proximal basal crease to the tip.

To gather sociodemographic information, an interviewer‐administered semi‐structured questionnaire was employed. Measurements of standing height and body weight were conducted with precision, using a stadiometer and body weighing scale, respectively, accurate to 0.1 cm and 0.1 kg. The body mass index (BMI) was then computed as body weight divided by the square of the height (Kg/m^2^). For finger length assessments, scans of the hands were subjected to computer‐assisted analysis, following the methodology outlined by Fink and Manning in 2018. The lengths of the second and fourth fingers on both hands were measured from the most proximal basal crease to the tip of the finger, as described by Fink and Manning (2018). Each finger was measured twice by the same observer, and the averaged values were used for analysis (see Figure 1). The second‐to‐fourth digit ratios for the right (2D:4DR) and left (2D:4DL) hands were calculated, along with the right–left 2D:4D difference (Dr‐l). To ensure measurement reliability, intraclass correlation coefficients (ICC) between the repeated measurements were calculated using the two‐way mixed, single measures with absolute agreement technique. The ICC values were 0.977 and 0.980 for 2D:4DL and 2D:4DR, respectively. Venous blood samples were obtained from each participant using gel‐separator tubes. After clotting at 4–8°C, the samples were centrifuged at 1500 rpm for 10 minutes to obtain serum. The serum samples were aliquoted and stored at −20°C without thawing or refreezing until analysis. Serum testosterone levels were determined using the ELISA technique (Monobind Inc., Lake Forest, CA 92630, USA). All sample collections were performed between 8:00 am and 12:00 pm local time to minimize diurnal variability.

### 2.3 Statistical analysis

After recording the data in an Excel spreadsheet, it was transferred to SPSS (version 27) for thorough analysis. The normality of continuous data distribution was examined using the Shapiro–Wilk test. Categorical data were presented as frequencies (percentage), while continuous variables were expressed as mean ± standard deviation. The chi‐square or Fisher's exact test was applied to evaluate disparities in data distribution among groups for categorical variables. Additionally, the unpaired *t*‐test (two‐tailed) was utilized to compare continuous data distribution between groups in the case of two groups. However, the unpaired *t*‐test (1‐tailed) was used to compare the serum total testosterone, the 2D:4D ratio and the Dr‐l because males and females of OS twin pairs are hypothesized to have higher total testosterone but lower digit ratios when compared to females of SS twin pairs. Since SPSS does not have a one‐tailed *t*‐test, a two‐tailed *t*‐test was performed and the *p* value was then halved. The effect size was presented as Hedges' g due to differences in sample size between groups. Statistical significance for differences between groups was established at a *p* value <0.050.

### 2.4 Ethical declaration

This study adhered to the principles outlined for human subject research in the 1964 Declaration of Helsinki and its subsequent revisions. Approval for the study was granted by the Institutional Review Board of the University for Development Studies. Before sampling, written informed consent was obtained from all participants, affirming their voluntary participation and the freedom to withdraw from the study at any stage. The research embraced inclusivity, transcending limitations based on religious, cultural, or political affiliations.

## 3 RESULTS

### 3.1 Sociodemographic profile of study participants

Table 1 provides a concise overview of the sociodemographic characteristics of the study population. A significant proportion (65%) of participants identified with the Mole‐Dagomba cultural group, while the majority adhered to the Islamic faith. The categorization of the twins into cultural groups and religious affiliations is a matter of convenience and not based on any criteria that may markedly affect the results. Ghanaians identify with various cultural or religious groups largely based on sociological and not genetic or anthropometric factors.

**TABLE 1:** The sociodemographic characteristics of the study population.

Variable	Frequency	Percent
Sex		
Male (OS)	10	25.0
Female (OS)	10	25.0
Female (SS)	20	50.0
Cultural group		
Mole‐Dagomba	26	65.0
Others	14	35.0
Religious affiliation		
Christianity	17	42.5
Islam	23	57.5

*Note*: The results are summarized as frequency and percentages.

Abbreviations: OS, opposite‐sex; SS, same‐sex.

### 3.2 Sex differences

As depicted in Table 2, the serum testosterone levels were notably higher in male of OS twin pairs compared to the combined female twins (*p* < 0.001). Additionally, males of OS twin pairs exhibited greater length in both second (2D) and fourth (4D) fingers (digits) on both hands than the combined female twins. However, the 2D:4D ratio did not exhibit a statistically significant difference between male of OS twin pairs and the combined female twins.

**TABLE 2:** Comparison variables between males and females.

Variable	Male (OS)	Female (all)	*t*	*p* Value	Hedges' *g*
Age (years)	21 ± 3	22 ± 3	−0.866	0.392	−0.31
BMI (Kg/m^2^)	21.1 ± 2.6	22.3 ± 3.2	−1.079	0.288	−0.39
TT (nmol/L)^a^	4.6 ± 1.6	0.6 ± 0.3	13.087	<0.001	4.68
2DR (cm)	7.49 ± 0.46	6.80 ± 0.52	3.738	0.001	1.34
4DR (cm)	7.79 ± 0.44	7.11 ± 0.50	3.879	<0.001	1.39
2DL (cm)	7.50 ± 0.68	6.86 ± 0.59	2.889	0.006	1.03
4DL (cm)	7.90 ± 0.56	7.09 ± 0.48	4.469	<0.001	1.60
2D:4DR^a^	0.961 ± 0.031	0.957 ± 0.044	0.250	0.402	0.09
2D:4DL^a^	0.949 ± 0.028	0.967 ± 0.047	−1.192	0.120	−0.43
Dr‐l^a^	0.012 ± 0.033	−0.010 ± 0.057	1.184	0.122	0.42

*Note*: The results are summarized as mean ± SD. The differences between males of OS and all female twins were determined using the unpaired student *t*‐test.

Abbreviations: 2DR, second digit; 4D, fourth digit; Dr‐l, the right‐ and left‐hand difference in digit ratio; L, left hand; OS: opposite‐sex; R, right hand; TT, total testosterone.

^a^ One‐tailed *t*‐test was used to determine differences between groups.

### 3.3 Disparities between females of same‐sex and females of opposite‐sex twin pairs

Table 3 illustrates the comparison between variables for females of opposite‐sex and females of same‐sex twins pairs. No significant differences were observed in the lengths of the second (2D) or fourth (4D) digits, as well as the 2D:4D ratio, for both hands. However, females of SS twin pairs exhibited a norminally higher serum testosterone level compared to females of OS twin pairs (*p* = 0.013).

**TABLE 3:** Comparison of variables between same‐ and opposite‐sex female twins.

Variable	Females (OS)	Females (SS)	*t*	*p* Value	Hedges' *g*
Age (years)	21 ± 3	22.3 ± 2.7±	−1.269	0.215	−0.478
BMI (Kg/m^2^)	22.0 ± 2.7	22.5 ± 3.4	−0.413	0.683	−0.156
TT (nmol/L)^a^	0.4 ± 0.1	0.67 ± 0.34	−2.649	<0.001	−0.998
2DR (cm)	7.01 ± 0.56	6.69 ± 0.48	1.642	0.112	0.619
4DR (cm)	7.32 ± 0.59	7.00 ± 0.43	1.627	0.115	0.613
2DL (cm)	7.13 ± 0.68	6.72 ± 0.50	1.881	0.070	0.709
4DL (cm)	7.38 ± 050	6.94 ± 0.41	2.552	0.016	0.962
2D:4DR^a^	0.960 ± 0.049	0.955 ± 0.042	0.301	0.383	0.113
2D:4DL^a^	0.966 ± 0.048	0.968 ± 0.047	−0.117	0.454	−0.044
Dr‐l^a^	−0.005 ± 0.080	−0.013 ± 0.043	0.328	0.373	0.124

*Note*: The results are summarized as mean ± SD. The differences between groups were determined using an unpaired *t*‐test.

Abbreviations: 2DR, second digit; 4D, fourth digit; Dr‐l, the right‐ and left‐hand difference in digit ratio; L, left hand; OS, opposite‐sex; R, right hand; SS, same‐sex; TT, total testosterone.

^a^ One‐tailed *t*‐test was used to determine differences between groups.

### 3.4 Multiple testing corrections

Multiple testing corrections was done to reduce the false positive rate due to multiple hypothesis testing using the Bonferroni multiple testing methods (Table 4). It was observed that the serum total testosterone level was higher in males of opposite‐sex twin pairs than either females of same‐sex (*p* < 0.001) or females of opposite‐sex twin pairs (*p* < 0.001). However, the difference in the 2D:4D ratio and left–right 2D:4D difference were insignificant between groups despite the observed significant differences in digit lengths.

**TABLE 4:** Multiple testing corrections using the Bonferroni multiple comparison technique.

Variables	Male (OS)	Females (OS)	Females (SS)	*p* Value
Age (years)	21 ±3	21 ± 3	22.3 ± 2.7	0.331
BMI (Kg/m^2^)	21.1 ± 2.6	22.0 ± 2.7	22.5 ± 3.4	0.522
TT (nmol/L)	4.6 ± 1.6	0.4 ± 0.1^##^	0.67 ± 0.34^##^	<0.001
2DR (cm)	7.49 ± 0.46	7.01 ± 0.56	6.69 ± 0.48^##^	0.001
4DR (cm)	7.79 ± 0.44	7.32 ± 0.59	7.00 ± 0.43^##^	0.001
2DL (cm)	7.50 ± 0.68	7.13 ± 0.68	6.72 ± 0.50^#^	0.005
4DL (cm)	7.90 ± 0.56	7.38 ± 050	6.94 ± 0.41^##^	<0.001
2D:4DR	0.961 ± 0.031	0.960 ± 0.049	0.955 ± 0.042	0.921
2D:4DL	0.949 ± 0.028	0.966 ± 0.048	0.968 ± 0.047	0.503
Dr‐l	0.012 ± 0.033	−0.005 ± 0.080	−0.013 ± 0.043	0.480

^#^ *p* < 0.010, ^##^ *p* < 0.001 compared to male (OS).

## 4 DISCUSSION

The twin testosterone transfer (TTT) hypothesis has potential implications for establishing hematological and biochemical reference ranges specifically tailored to female twins. This study investigated whether females of opposite‐sex (OS) twin pairs exhibit a lower 2D:4D ratio but higher serum testosterone levels compared to females of same‐sex (SS) twin pairs. The findings revealed no significant distinctions neither in the 2D:4D ratio between males of OS twin pairs and all female twin pairs nor between females of OS and females of SS twin pairs for both hands. However, there was a notable discrepancy in testosterone levels, with males of OS twin pairs displaying significantly higher levels than all female twin pairs even after multiple testing correction analysis. Females of SS twin pairs exhibited significantly higher testosterone levels than their females of OS twin pairs counterparts; however, this observation was lost after multiple testing correction analysis.

The findings reveal no statistically significant difference in the second‐to‐fourth digit ratio (2D:4D) between females of opposite‐sex and females of same‐sex twin pairs. This observation aligns with the results reported by Medland, Loehlin, and Martin (2008) but contradicts the outcomes of previous studies (van Anders et al., 2006; Voracek & Dressler, 2007). In their study, Medland, Loehlin, and Martin (2008) examined a large sample of 867 females of same‐sex and females of opposite‐sex dizygotic twins. They concluded that although female's 2D:4D was higher than males' in both hands, females of same‐sex and females of opposite‐sex twin pairs did not differ significantly in their 2D:4D ratio. On the contrary, Voracek and Dressler (2007) and van Anders et al. (2006) found a significant difference in the 2D:4D ratio between females of same‐sex and females of opposite‐sex twins in a sample of 114 twin pairs (in the former study) and 58 twin pairs (in the latter study). There are differences in sample size between the previous studies as well as between the previous and the current study. Although the sample size may contribute to inter‐study variabilities, one suggested explanation for the lack of difference in 2D:4D between males of OS and female twin pairs may be that males of OS twin pairs might experience feminization or hypo‐masculinization compared to singleton males due to exposure to prenatal oestrogen from their female co‐twins. However, limited evidence supports this hypothesis (Cohen‐Bendahan et al., 2004).

Regarding females of OS twin pairs, a plausible explanation is that the level of prenatal testosterone exposure for females of OS twin pairs from their male co‐twins may be insufficient to exert a significant effect. This argument is supported by the lack of genital virilization in females of OS twin pairs, in contrast to females with congenital adrenal hyperplasia (CAH) caused by 21‐hydroxylase deficiency, who are exposed to excess prenatal testosterone (Cohen‐Bendahan et al., 2004). Additionally, it appears that prenatal testosterone exposure may not occur at the critical period of fetal development when it would have had a significant impact on the 2D:4D ratio which is likely to be the period before the 14th week of pregnancy (Zheng & Cohn, 2011).

The TTT and the 2D:4D ratio have been a matter of controversy among scholars. It is argued that the application of the twin testosterone transfer concept to humans is conjectural and draws inspiration from evidence found in nonhuman mammalian studies. Some argue that robust evidence supporting TTT is primarily observed when a nonhuman mammalian female foetus is gestated between or in proximity to two male foetuses (Ahrenfeldt et al., 2015). Moreover, the designation of the 2D:4D ratio as the presumed marker of prenatal testosterone (PT) exposure is a matter of debate. Regarding the 2D:4D ratio, it is critiqued to be an artifact of allometry; that the difference in digit ratio between males and females is caused by a shift in the common allometric line with nonzero intercept, an indication that 2D:4D decreases with increasing finger lengths. Since men have larger hands and longer fingers, they tend to have a lower 2D:4D ratio (Kratochvíl & Flegr, 2009; Leslie, 2019; Lolli et al., 2017). However, despite prior investigations reporting no or weak significant sex differences in digit ratios, (Marczak et al., 2018), a meta‐analysis of available data has proved the validity of the 2D:4D ratio (Hönekopp & Watson, 2010). An earlier suggestion was made, particularly for males, that prenatal Leydig cell activity was correlated with that of adult Leydig cell activity. In that regards, the 2D:4D, a marker of prenatal testosterone exposure will be inversely associated with circulating levels of testosterone in adulthood. However, previous studies including meta‐analysis have concluded that there is no correlation, or where correlation exists, the effect size is small or negligible (de Sanctis et al., 2017; Hönekopp et al., 2007; Muller et al., 2011). This study, to the best of our knowledge, is likely the first attempt within an African population to explore evidence of the twin testosterone transfer (TTT) phenomenon in the 2D:4D ratio. Additionally, the lengths of fingers were acquired from hand scans and subsequently measured using computer‐assisted analysis, a methodology deemed more precise than direct or alternative indirect techniques, such as measurements from photocopies (Fink & Manning, 2018). In addition, multiple testing correction analysis was done to protect against false positives. However, it is important to note that the study is exploratory due to its relatively smaller sample size in comparison to earlier investigations (Medland, Loehlin, & Martin, 2008). The sample size is most important as, typically, sex differences for 2D:4D are of small‐to‐medium effect size. It was, however, challenging to find enough number of twins to participate in the study. The lack of zygosity differentiation between the female twin pairs is another limitation of the study. However, the true value of this study lies in the observation that there is a lack of existing research in non‐European countries regarding the TTT and the 2D:4D in twin populations. The study sought to address the apparent limited ancestral diversity in TTT and 2D:4D research. The authors will recommend further studies involving a larger sample size.

## 5 CONCLUSION

This study revealed no notable distinctions in the second‐to‐fourth digit (2D:4D) ratio and serum testosterone levels between females of opposite‐sex and females of same‐sex female twin pairs. These findings suggest that the twin testosterone transfer phenomenon may either not occur, or its influence on digit ratios and testosterone levels might be too subtle to yield significant effects.

## AUTHOR CONTRIBUTIONS

Moses Banyeh and Augusta S. Kolekang: Project administration, supervision, validation, statistical analysis and writing‐draft. Moses Banyeh, Martha Nyewie, Abul‐Rafik Abdulai, Charles Nkansah, Gabriel Abbam, Thea Kangkpi, Kervin Edinam Zogli, and Shafiat Omotoyosi Shittu: conceptualization and methodology. David Bure, Romarick Kofi Wemegah, Mikail Ihasan Azindow, Suleman Yakubu, Musah Seidu, Mohammed Madde Baba, Elisha Essoun, and Nancy Owireduwaa: experimentation and data collection. All authors reviewed the work and approved the final version of the manuscript. The corresponding author has full access to all of the data in this study and takes complete responsibility for the integrity of the data and the accuracy of the data analysis.

## FUNDING INFORMATION

This research did not receive any specific grant from funding agencies in the public, commercial, or not‐for‐profit sectors.

## CONFLICT OF INTEREST STATEMENT

The authors have no competing interests to declare.

## Data Availability

The data supporting the results can be obtained from the corresponding author upon reasonable request.
